# Influence of Strategic Cortical Infarctions on Pupillary Function

**DOI:** 10.3389/fneur.2018.00916

**Published:** 2018-10-29

**Authors:** Costanza Peinkhofer, Pernille Martens, Johannes Grand, Thomas Truelsen, Gitte M. Knudsen, Jesper Kjaergaard, Daniel Kondziella

**Affiliations:** ^1^Department of Neurology, Copenhagen University Hospital Rigshospitalet, Copenhagen, Denmark; ^2^Neurobiology Research Unit, Rigshospitalet, Copenhagen, Denmark; ^3^Medical Faculty, University of Trieste, Trieste, Italy; ^4^Department of Radiology, Copenhagen University Hospital Rigshospitalet, Copenhagen, Denmark; ^5^Department of Cardiology, Copenhagen University Hospital Rigshospitalet, Copenhagen, Denmark; ^6^Faculty of Health and Medical Sciences, University of Copenhagen, Copenhagen, Denmark; ^7^Department of Neuroscience, Norwegian University of Science and Technology, Trondheim, Norway

**Keywords:** endovascular stroke therapy, insula, prefrontal eye field, pupils, pupillometry, pupillary light reflex, stroke, mechanical thrombectomy

## Abstract

**Objective:** Cortical activity, including cognitive and emotional processes, may influence pupillary function. The exact pathways and the site of cortical pupillary innervation remain elusive, however. We investigated the effects of select cortical strokes, i.e. ischemic infarcts affecting the insular cortex and prefrontal eye field, on pupillary function.

**Methods:** Seventy-four patients with acute ischemic stroke, consecutively admitted to our institution from March to July 2018, were assessed 24 h after endovascular recanalization therapy (i.e., day 2 after the stroke), using automated pupillometry. Stroke location and volume and clinical severity (estimated by the Alberta Stroke Program Early CT Score and National Institute of Health Stroke Scale) were recorded. We excluded patients with posterior circulation stroke, intracranial pathology other than ischemic stroke, midline shift on computed tomography exceeding 5 millimeters or a history of eye disease. Pupillometry data from 25 neurologically normal patients with acute myocardial infarction were acquired for control.

**Results:** Fifty stroke patients after thrombectomy were included for analysis. Twenty-five patients (50%) had insular cortex or prefrontal eye field involvement (group 1, strategic infarcts); 25 patients had infarcts located in other cerebral areas (group 2, other infarcts). The pupillary light reflex, as measured by constriction velocity and maximal/minimal pupillary diameters, was within physiological limits in all patients, including controls. However, while pupillary size and constriction velocities were correlated in all subjects, the correlation of size and dilatation velocity was absent in right-hemispheric infarcts (left hemisphere infarcts, group 1 (*r*^2^ = 0.15, *p* = 0.04), group 2 (*r*^2^ = 0.41, *p* = 0.0007); right hemisphere infarcts, group 1 (*r*^2^ = 0.008, *p* = 0.69); group 2 (*r*^2^ = 0.12, *p* = 0.08); controls (*r*^2^ = 0.29, *p* ≤ 0.0001).

**Conclusions:** Cortical infarcts of the prefrontal eye field or insula do not impair the pupillary light reflex in humans. However, subtle changes may occur when the pupils dilate back to baseline, probably due to autonomic dysfunction. Replication is needed to explore the possible influence of hemispheric lateralization. We suggest that endovascular therapy for acute ischemic stroke may serve as a clinical research model for the study of acquired cortical lesions in humans.

## Introduction

The pupillary light reflex is a polysynaptic reflex that requires cranial nerves II and III, as well as central brainstem connections (1). Light falling into one eye stimulates retinal photoreceptors, bipolar cells and subsequently retinal ganglion cells, whose axons form the optic nerve. Some of these axons terminate in the pretectum of the mesencephalon; and pretectal neurons project further to the Edinger-Westphal nuclei. Then, preganglionic parasympathetic axons synapse with ciliary ganglion neurons which in turn send postganglionic axons to innervate the pupillary constrictor muscles of both eyes (1).

Although the pupillary light reflex is part of the routine neurological examination, its physiological background is less well-understood than most clinicians are aware of. In addition to the pathways outlined above, there is also a cortical component of pupillary innervation. For instance, emotional responses and cognitive processes such as decision making and mental arithmetic may produce pupillary dilatation (2–8). Further, electrical stimulation of the frontal eye field in monkeys leads to pupillary dilatation (9, 10). Via connections with the intermediate layer of the superior colliculus, the frontal eye field appears to be able to modulate pupillary diameters, resulting in pupillary dilation during cognitive processes (10). Another gray matter region that may contribute to pupillary function is the insular cortex. An important region for arousal and autonomic control (11, 12), the insular cortex is involved [together with the anterior cingulate cortex (13, 14)] in the control of the locus coeruleus, the noradrenergic brainstem center (15), and may thereby influence pupillary function via sympathetic-parasympathetic innervation (13, 16, 17). However, the exact pathways of cortical modulation of human pupillary function remain elusive.

The present study aimed at investigating cortical modulation of pupillary reflex pathways by using routinely collected data in a clinical setting. To this end, we correlated automated pupillometry with cerebral infarct locations in stroke patients after endovascular thrombectomy, which served as a paradigm for pupillary changes caused by select cerebral lesions. We hypothesized that patients with strategic infarcts localized to the prefrontal eye field (Broadman area 8) and/or the insular cortex on either side would have pupillary abnormalities compared to stroke patients without infarcts in these areas. Pupillometry data from neurologically normal patients with acute but clinically stable myocardial infarction, investigated after percutaneous coronary intervention, served as control group.

## Materials and methods

### Inclusion criteria

We assessed pupillometry data from stroke patients (aged ≥18 years) with an anterior circulation stroke (i.e., affecting internal carotid artery, middle cerebral artery and/or anterior cerebral artery territories) consecutively admitted for acute endovascular thrombectomy to the Department of Neurology, Rigshospitalet, Copenhagen University Hospital, during the period from March to July 2018.

### Exclusion criteria

Patients with a history of eye disease (e.g., following cataract operation), relevant structural pathology on CT other than ischemic stroke (e.g., tumors), and mass effects on CT exceeding a midline shift of 5 mm (measured at the level of the pineal gland) were excluded. In addition, we excluded patients with evidence of posterior circulation strokes (acute or chronic) to avoid lesions involving the brainstem and occipital cortex.

### Procedures

**Figure 1:**
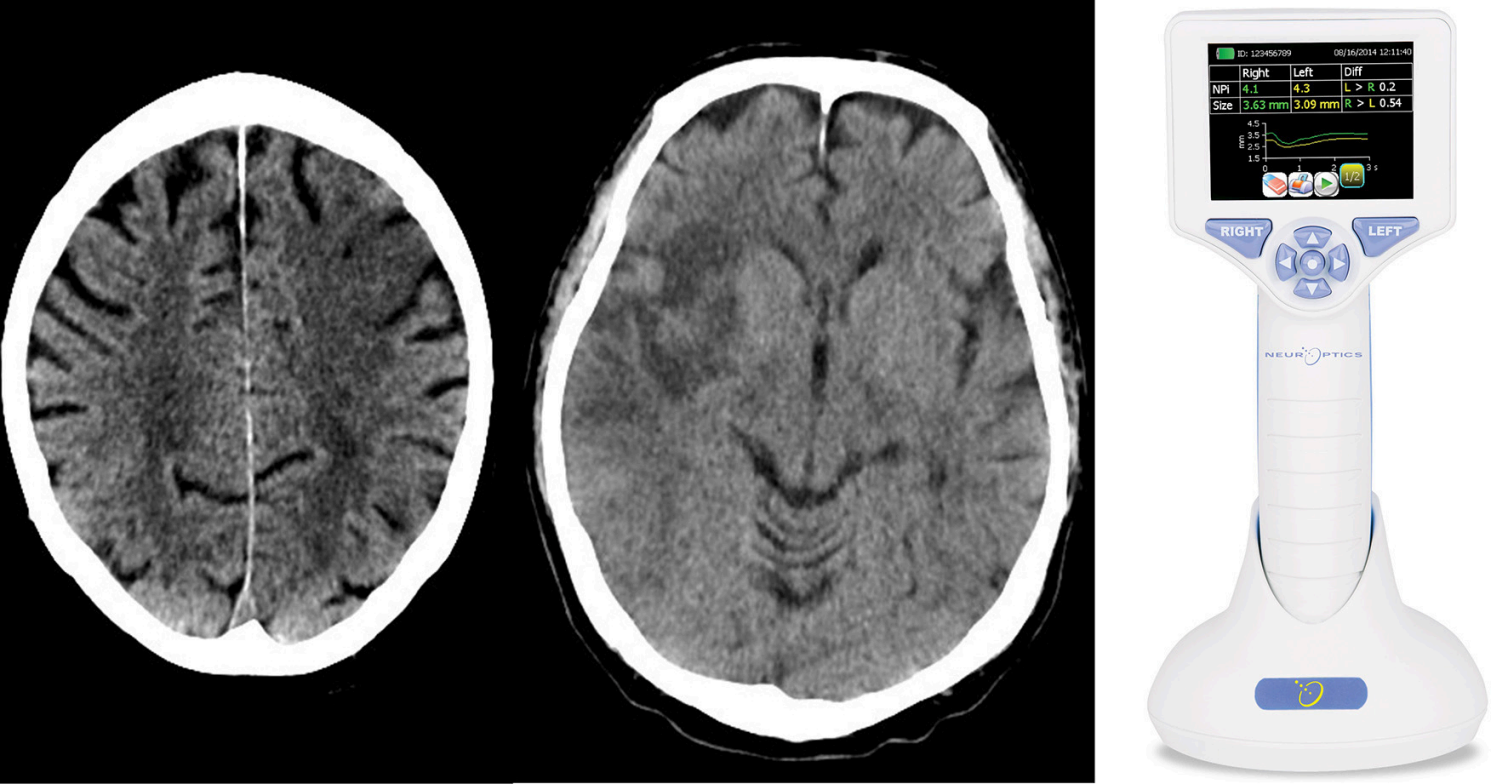
We performed a clinical practice study investigating the cortical modulation of pupillary function following strategic cerebral strokes. This figure depicts CT of the brain from 2 exemplary stroke patients 24 h following endovascular therapy for large vessel occlusive stroke. Strategic ischemic infarctions are seen in the left prefrontal eye field **(Left)** and right insular cortex **(Central)**. Using automated pupillometry [**(Right)**; courtesy of https://commons.wikimedia.org/wiki/Main_Page], we collected pupillometry data of patients with strategic infarcts in the prefrontal eye field and/or insular cortex (group 1) and compared them to data from stroke patients without infarcts in these areas (group 2) and to data from patients with myocardial infarcts but without clinical evidence of brain injury (control group).

For automated pupillometry, we used the NPi®-200 Pupillometer (NeurOptics, Laguna Hills, CA 92653 USA), a portable, handheld, monocular, infrared device, which allows quantitative measurements of the pupillary response. The pupillometer releases a flash of white light (duration 0.8 s, pulse intensity 121 uW) to stimulate the pupillary light reflex. Light calibration is performed by the manufacturer and does not require any periodic re-calibration. The pupillometer digitally registers the pupillary light response as a video (sampling rate 30 Hz) and displays numeric results on a screen (Table 1, Figure 1). For an illustration of the NPi®-200 pupillometer, please consider https://www.youtube.com/watch?v=EjlZ5oocl0g&frags=pl%2Cwn. Measurements were performed once in both eyes as part of the routine clinical evaluation 24 h after the endovascular treatment (i.e., on the second day of stroke) immediately before the CT scan control. During pupillometry measurement of each eye the opposite eye was covered to minimize the consensual light reflex and its effect on the pupillary baseline diameter. National Institute of Health Stroke Scale (NIHSS) scores 24 h after stroke onset were also collected as part of the clinical routine. Twenty-four hours control computed tomography (CT) of the brain was assessed by a trained neuroradiologist for infarctions localized in the prefrontal eye field (Brodmann area 8), the insular cortex and/or the thalamus in either hemisphere (Figure 1). In addition, CT was evaluated for overall stroke volume using the Alberta Stroke Program Early CT Score (ASPECTS) (18). We also recorded whether infarcts occurred in the thalamus, as this region is an important relay station of the visual pathway (19) (although typically supplied by the posterior, not anterior, circulation).

**Table 1:** Variables assessed by pupillometry.

Size = Maximal Diameter (in millimeters)	Maximum pupil size before constriction
MIN = Minimal Diameter (in millimeters)	Pupil diameter at peak constriction
% CH = Change in diameter (%)	% of change from maximal to minimal pupil diameter
LAT = Latency of constriction (in seconds)	Time of onset of constriction following initiation of the light stimulus
CV = Constriction Velocity (in millimeters per second)	Average of how fast the pupil diameter is constricting measured in millimeters per second
MCV = Maximum Constriction Velocity (in millimeters per second)	Maximum velocity of pupil constriction of the pupil diameter responding to the flash of light measured in millimeters per second
DV = Dilation Velocity (in millimeters per second)	The average pupillary velocity when, after having reached the peak of constriction, the pupil tends to recover and to dilate back to the initial resting size, measured in millimeters per second
NPi = Neurological Pupil Index (absolute value)	Proprietary algorithm that takes all variables above as inputs and compares to normative model to give a composite score of pupillary response from 0 to 5, with ≥3 being within physiological limits (21)


Pupillometry, CT, and NIHSS data were dichotomized according to stroke location: Group 1 included stroke patients with infarcts in the prefrontal eye field and insular cortex in either hemisphere (strategic infarcts); group 2 included stroke patients without infarcts in these regions. The control group consisted of patients with acute, clinically stable myocardial infarction after percutaneous coronary intervention from the Department of Cardiology, Rigshospitalet, Copenhagen University Hospital. The latter patients underwent pupillometry as described, but neither NIHSS nor CT.

### Outcome measures

Outcome measures included the pupillary diameter before and after light exposure, percentage change of pupillary diameters, and pupillary constriction and dilatation velocities, as well as the neurological pupil index (NPi), which is a proprietary pupillometry sum score (i.e., a composite of quantitative pupillary parameters and a measure of the briskness of the pupil light reflex) from 0 to 5, with ≥3 indicating physiological limits (including a maximal difference between the 2 eyes of < 0.7) (20, 21) (Table 1). In addition, strategic infarcts were evaluated as outlined above (Figure 1).

### Statistics

Statistical tests were performed using Prism 7 software (GraphPad Software; La Jolla, CA, USA). Baseline characteristics, dichotomized ASPECT (18) scores and NIHSS (22) and all pupillary parameters were first compared between group 1 (strategic strokes) and group 2 (other strokes), and then between group 1 and controls using either Fisher's exact test or unpaired or paired two tailed Student's *t*-tests. Where required, the Holm-Sidak method was performed to adjust results for multiple testing. In addition, the relationship between constriction and dilation velocities, respectively, maximum size before constriction and minimum size after constriction (i.e., diameter at maximal constriction) was examined using Pearson's correlation coefficient and linear regression for left and right eye in group 1 and controls; for left and right hemisphere infarcts in all three groups. A statistically significant difference was defined by a value of *p* < 0.05.

### Ethics

All measurements were performed as part of routine clinical assessment. Data were anonymized and handled according to the European Union's Data Protection Law. The Ethics Committee of the Capital Region of Denmark approved the study concept and waived the need for written consent because risks were deemed negligible.

## Results

### Patients

Seventy-four patients were admitted for acute endovascular thrombectomy from March 1 to July 11, 2018. Twenty-four patients fulfilled exclusion criteria and their data were omitted. Fifty patients with an anterior circulation stroke were included for analysis [26 (52%) females; mean age 71.8 years, *SD* ± 10.8]. All stroke patients received endovascular treatment, followed by a control CT, NIHSS score and an assessment of pupillary function 24 h later. The control group consisted of 25 age- and sex-matched patients [11 (44%) females; mean age 67.8 years, *SD* ± 13.6] with acute, clinically stable myocardial infarction and without known cerebral stroke. Clinical baseline data are provided in Table 2. For raw data (clinical, radiological and pupillometric data) see Supplementary Table 1.

**Table 2:** Clinical baseline characteristics.

	**Group 1**	**Group 2**	**Controls**	*p*-value^*^	*p-*value^**^
	**Stroke patients with strategic infarctions (*n* = 25)**	**Stroke patients without strategic infarctions (*n* = 25)**	**Patients with myocardial infarction (*n* = 25)**	
Age (years; mean ± standard deviation)	72.6 ± 11.8	71.9 ± 8	67.8 ± 13.6	NS	NS
Females	12 (48%)	14 (56%)	11 (44%)	NS	NS
Hypertension	23 (92%)	20 (80%)	15 (60%)	NS	0.009
Cholesterol	19 (76%)	21 (84%)	23 (92%)	NS	NS
Diabetes	5 (20%)	4 (16%)	2 (8%)	NS	NS
Smoking	7 (28%)	8 (32%)	5 (20%)	NS	NS
Alcohol Abuse	4 (16%)	3 (12%)	0	NS	NS
Platelet Inhibition	18 (72%)	20 (80%)	24 (96%)	NS	NS
Anticoagulation	7 (28%)	5 (20%)	3 (12%)	NS	NS
Antihypertensives	20 (80%)	16 (64%)	14 (56%)	NS	NS
Sedative medication (i.e. antiepileptic and psychotropic drugs)	1 (4%)	6 (24%)	1 (4%)	NS	NS

*NS, not significant; significance level p < 0.05*;

* *Group 1 vs. group 2*;

** *Group 1 vs. controls*.

### Location of cerebral infarcts and stroke burden

Of 25 patients with strategic infarctions (group 1), 17 had an involvement of the insular cortex alone, 2 of the prefrontal eye field, and 5 of both areas (Figure 1). Only 1 patient had a thalamic infarct, together with both insular and prefrontal eye field lesions. Eleven patients (44%) had a left hemispheric stroke, 14 (56%) a right-side hemispheric stroke. Twenty-five patients had lesions in other brain areas, 12 (48%) had a left sided stroke, 13 (52%) a right hemispheric stroke (group 2).

As expected, infarct volumes (estimated with the ASPECT score) were correlated with the presence of a strategic stroke. Patients with an ASPECT score ≤ 7 (*n* = 20 or 80% in group 1; *n* = 3 or 12% in group 2), indicating higher stroke volumes, had a significantly higher chance of having an insular or prefrontal eye field involvement compared to those with ASPECT >7 (Fisher's exact test; *p* < 0.0001).

The clinical severity, as revealed by the NIHSS score, was also associated with stroke location. NIHSS scores >10 (*n* = 15 or 56% in group 1; *n* = 6 or 24% in group 2) correlated with a higher probability of strategic infarctions (Fisher's exact test; *p* = 0.01).

### Pupillometry

General pupillary function was normal in the 3 groups: The NPi index was >3 in all 75 patients (i.e., 150 eyes examined). Likewise, NPi differences between left and right eyes were always within physiological limits (< 0.7). Maximal and minimal pupillary diameters, percentage changes in pupillary sizes, latency of pupillary constrictions, as well as constriction and dilation velocities were also similar between group 1 (strategic infarcts) and group 2 (other infarcts) (Table 3). There were neither any differences of these parameters between group 1 and controls following adjustment for multiple testing (Table 3). In addition, the relative amplitude (initial diameter–minimum size) of the pupillary light reaction was calculated for group 1 and compared between left and right eyes (paired *t*-test *p* = 0.25, effectiveness of pairing r = 0.63, *p* = 0.0003), indicating normal consensual pupillary reactions.

**Table 3:** Pupillometry data from stroke patients with and without strategic infarctions, and controls.

	**Group 1**	**Group 2**	**Controls**	*p*-value^*^	*p*-value^**^
	**Stroke patients with strategic infarctions (*n* = 25)**	**Stroke patients without strategic infarctions (*n* = 25)**	**Patients with myocardial infarction (*n* = 25)**	
Npi L	4.52 ± 0.08	4.38 ± 0.08	4.42 ± 0.07	0.2	0.36
Size L (mm)	3.44 ± 0.16	3.31 ± 0.15	3.17 ± 0.17	0.55	0.24
Min L (mm)	2.33 ± 0.01	2.41 ± 0.09	2.30 ± 0.09	0.53	0.32
%Ch L	31.6 ± 1.71	26.1 ± 1.63	25.4 ± 2.07	0.03^†^	0.02^†^
CV L (mm/s)	2.09 ± 0.17	1.87 ± 0.16	1.71 ± 0.17	0.36	0.13
MCV L (mm/s)	3.39 ± 0.28	2.75 ± 0.25	2.59 ± 0.26	0.1	0.05^†^
DV L (mm/s)	0.88 ± 0.06	0.84 ± 0.08	0.80 ± 0.07	0.68	0.43
Lat L (s)	0.24 ± 0.01	0.24 ± 0.01	0.24 ± 0.01	0.54	0.84
Npi R	4.45 ± 0.08	4.44 ± 0.08	4.44 ± 0.07	0.99	0.94
Size R (mm)	3.34 ± 0.14	3.29 ± 0.17	3.17 ± 0.18	0.81	0.45
Min R (mm)	2.33 ± 0.09	2.35 ± 0.11	2.3 ± 0.09	0.84	0.84
%Ch R	29.4 ± 2.14	27.7 ± 1.42	25.4 ± 2.03	0.53	0.18
CV R (mm/s)	2.03 ± 0.15	2.09 ± 0.14	1.66 ± 0.17	0.74	0.12
MCV R (mm/s)	3.19 ± 0.27	3.06 ± 0.23	2.53 ± 0.24	0.88	0.11
DV R (mm/s)	0.83 ± 0.08	0.83 ± 0.06	0.84 ± 0.09	0.94	0.89
Lat R (s)	0.24 ± 0.01	0.23 ± 0.01	0.24 ± 0.01	0.33	0.97

*Values are referring to mean±SEM; significance level p < 0.05; L, left; R, right; mm, millimeters; s, seconds; for other abbreviations see Table 1*;

* *Group 1 vs. group 2*;

** *Group 1 vs. controls*;

† *No longer significant after adjustment for multiple testing (%Ch L p = 0.026, adjusted p = 0.344; p = 0.025, adjusted p = 0.333; MCV L p = 0.0461, adjusted p = 0.507)*.

**Figure 2:**
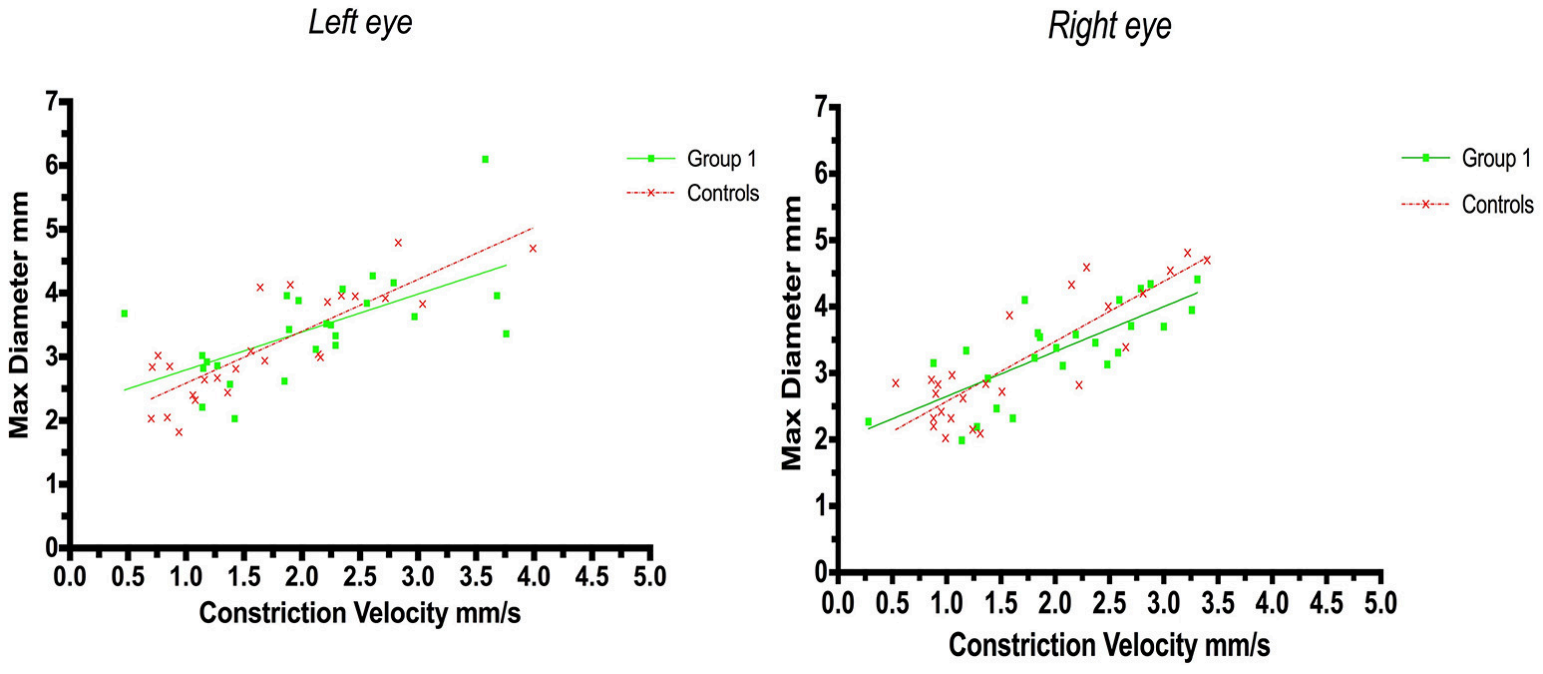
Pupillometry data from patients with strategic cerebral infarcts and controls: Maximal pupillary diameters and constriction velocities. Solid lines denote the best fit from linear regression analysis. **(Left)** Relationship between maximum diameter and constriction velocity of the left eye, group 1 in green (Y = 0.5945X+2.2, *r*^2^ = 0.38, *p* = 0.0009); controls in red (Y = 0.8142X + 1.772, *r*^2^ = 0.69, *p* < 0.0001). **(Right)** Relationship between maximum diameter and constriction velocity of the right eye, group 1 in green (Y = 0.6748X + 1.975, *r*^2^ = 0.57, *p* < 0.0001); controls in red (Y = 0.9028X +1.671, *r*^2^ = 0.72, *p* < 0.0001).

A positive correlation between maximum size and constriction velocity was found for both right and left eyes. The Pearson coefficient for left eyes was *r* = 0.62 (*p* = 0.0009) in group 1 (strategic infarcts) and *r* = 0.83 (*p* < 0.0001) in controls, and for right eyes the coefficient was *r* = 0.76 (*p* < 0.0001) in group 1 and *r* = 0.85 (*p* < 0.0001) in controls (Figure 2).

**Figure 3:**
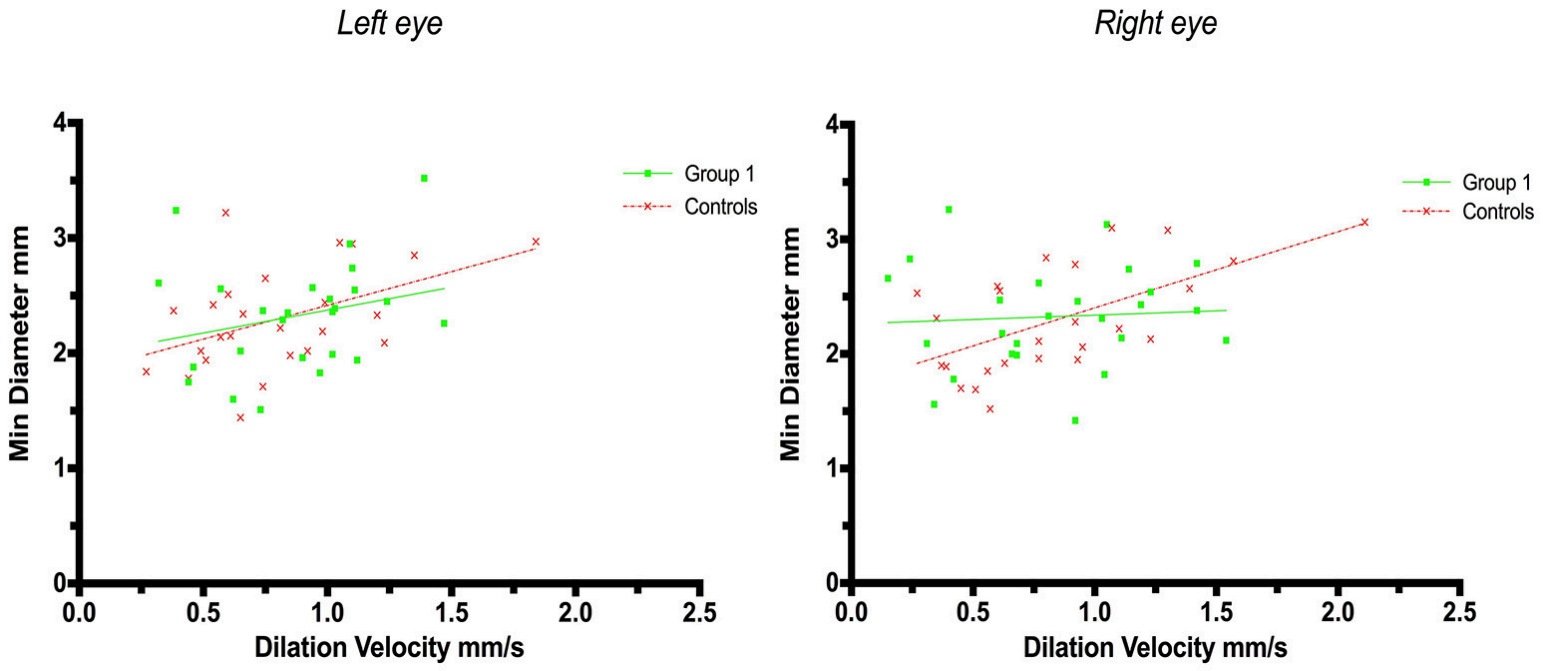
Pupillometry data from patients with strategic cerebral infarcts and controls: Minimal pupillary diameters and dilatation velocities. Solid lines denote the best fit from linear regression analysis. **(Left)** Relationship between minimum diameter and dilatation velocity of the left eye, group 1 in green (Y = 0.3976X + 1.977, *r*^2^ = 0.063, *p* = 0.23); controls in red (Y = 0.5857X + 1.83, *r*^2^ = 0.22, *p* = 0.018, adjusted *p* = 0.054). **(Right)** Relationship between minimum diameter and dilation velocity of the right eye, group 1 in green (Y = 0.07548X + 2.263, *r*^2^ = 0.004, *p* = 0.75); controls in red (Y = 0.6646X + 1.738, *r*^2^ = 0.37, *p* = 0.0013, adjusted *p* = 0.005).

A weak correlation was also found for minimum size and dilation velocity in controls but not in patients with strategic infarctions. Thus, the coefficient for left eyes in group 1 was r = 0.25 (*p* = 0.23) and in controls r = 0.47 (*p* = 0.018, adjusted *p* = 0.054), and in group 1 for right eyes *r* = 0.065 (*p* = 0.75) and in controls *r* = 0.60 (*p* = 0.0013, adjusted *p* = 0.005) (Figure 3).

**Figure 4:**
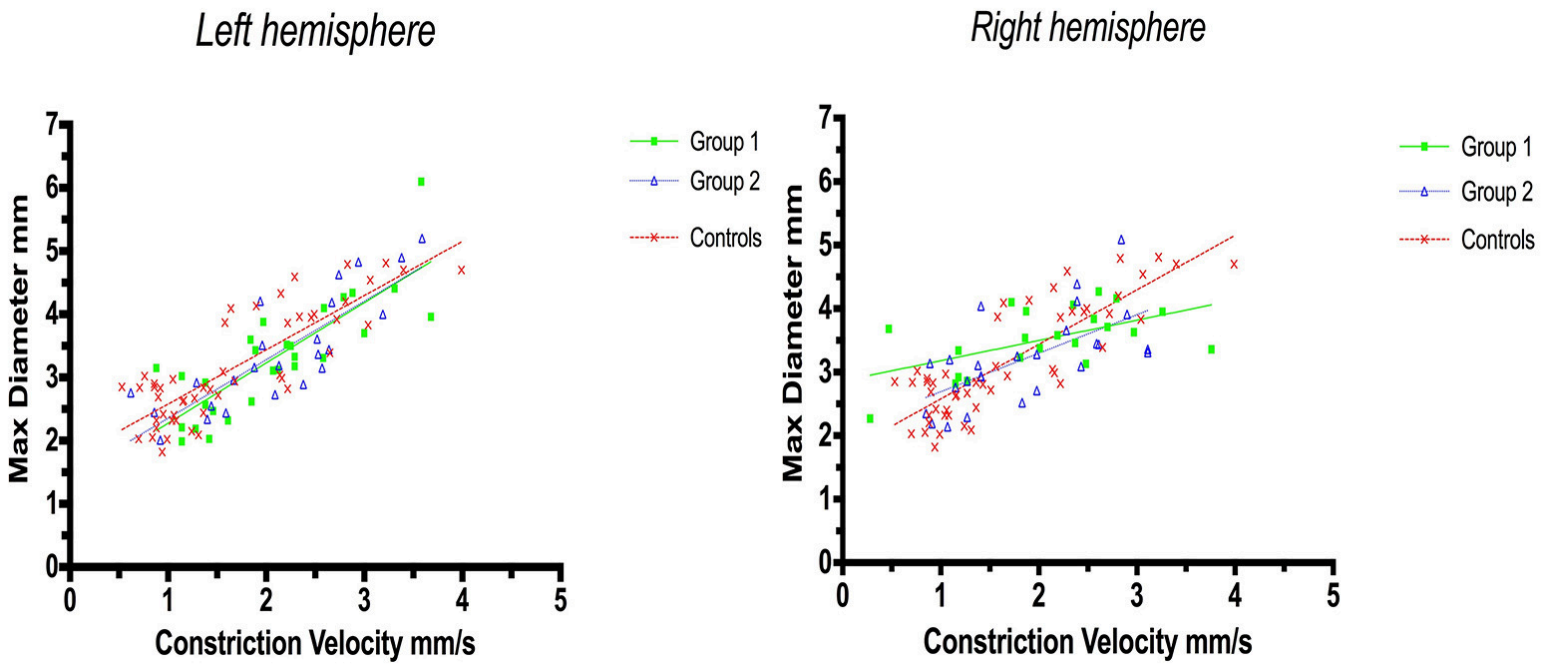
Pupillometry data from patients with left, respectively, right hemisphere cerebral infarcts (plotted against pupillometry data from controls without hemispheric strokes): Maximal pupillary diameters and constriction velocities. Solid lines denote the best fit from linear regression analysis. **(Left)** Left hemisphere infarcts, group 1 in green (Y = 0.9538X + 1.322, *r*^2^ = 0.67, *p* ≤ 0.0001); group 2 in blue (Y = 0.9257 X + 1.428, *r*^2^ = 0.7, *p* < 0.0001); controls in red (Y = 0.8583X + 1.721, *r*^2^ = 0.7, *p* ≤ 0.0001). **(Right)** Right hemisphere infarcts, group 1 in green (Y = 0.3189X + 2.861, *r*^2^ = 0.3, *p* = 0.0077); group 2 in blue (Y = 0.6064X + 2.086, *r*^2^ = 0.4, *p* = 0.0005), controls in red (identical values as above).

**Figure 5:**
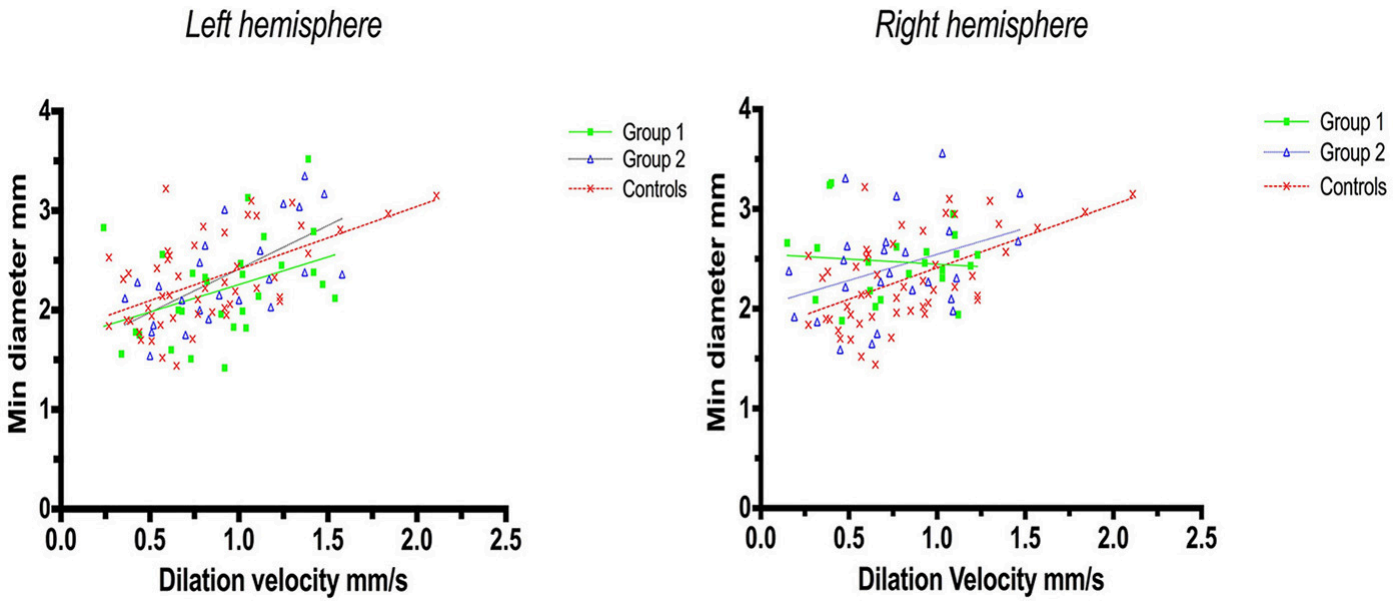
Pupillometry data from patients with left, respectively, right hemisphere cerebral infarcts (plotted against pupillometry data from controls without hemispheric strokes): Minimal pupillary diameters and dilatation velocities. Solid lines denote the best fit from linear regression analysis, showing the relationship between minimum diameter and dilation velocity. **(Left)** Left hemisphere infarcts, group 1 in green (Y = 0.5528X + 1.705, *r*^2^ = 0.15, *p* = 0.04); group 2 in blue (Y = 0.8756X + 1.538, *r*^2^ = 0.41, *p* = 0.0007); controls in red (Y = 0.6312 X 1.78, *r*^2^ = 0.3, *p* ≤ 0.0001). **(Right)** Right hemisphere infarcts, group 1 in green (Y = −0.1018 X+2.549, *r*^2^ = 0.008, *p* = 0.69); group 2 in blue (Y = 0.5222X + 2.023, *r*^2^ = 0.12, *p* = 0.0821), controls in red (identical values as above).

Linear regression analysis of constriction velocities, plotted against maximum pupillary diameters, was unaffected by hemispheric stroke lateralization (Figures 4, 5), but the correlation of dilatation velocities with minimal pupillary diameters was lost with strategic infarcts in the right hemisphere [group 1 (*r*^2^ = 0.008, *p* = 0.69); group 2 (*r*^2^ = 0,12, *p* = 0.0821); controls (*r*^2^ = 0.29, *p* ≤ 0.0001)] (Figure 5).

## Discussion

The human pupillary light reflex, as assessed by the speed of pupillary constriction and diameters before and after constriction, does not seem to be affected by strategic infarcts of the prefrontal eye field or insular cortex. This finding does not support the hypothesis of strategic strokes altering pupillary function, probably because the present model is a model of cortical lesioning as opposed to cortical activation. Hence, cortical activation may lead to pupillary dilation (5, 9) [or, more rarely, constriction (23–26)], but the absence of cortical input to the pupils following cortical damage does not appear to affect the light reflex. To our knowledge, this is the first systematic human study investigating cortical modulation of pupillary function in a true-to-life clinical setting.

Subtle changes in pupillary function, however, may still be possible immediately after the light reflex, i.e., when the light stimulus is over, and the pupils dilate back to baseline. Thus, while we observed a robust correlation between pupillary size and constriction velocity [confirming previous studies (27, 28)], minimum size and dilation velocity were still correlated in controls but no longer in patients with strategic infarctions. In addition, in patients with strategic infarcts the correlation of dilatation velocities with pupillary diameters was weak with left hemispheric strokes and lost with right-sided strokes. In contrast, this correlation was still robust in controls and in stroke patients without strategic infarcts, irrespective of hemispheric lateralization (Figure 4). Several explanations are possible. First, strategic infarctions in the prefrontal eye field and/or insular cortex may indeed influence pupillary function in subtle ways, perhaps by impaired sympathetic control or reduced parasympathetic inhibition, resulting in decreased pupillary dilation velocity (27). This is consistent with the insular cortex being an important center of autonomic control. Indeed, strokes affecting the insular cortex are associated with significant autonomic dysfunction (11, 12, 29, 30), in particular ischemic stroke events involving the right-sided insular cortex (31). Second, the observed difference could be related to infarct volume. Patients with strategic infarcts had larger stroke volumes and more severe neurological deficits compared to patients without strategic infarcts, and, although strokes producing mass effects were excluded, this may have influenced pupillary function either by accumulative neuronal loss or damage to still unidentified neuronal groups that may be important for pupillary function. Third, the results may be flawed due to the relatively small sample size. However, if these findings can be replicated with larger numbers, loss of cortical innervation would, indeed, seem to produce subtle pupillary changes as we excluded strokes with mass effect and posterior circulations strokes, i.e., the occipital visual areas and the subcortical neuronal innervation of the pupils were intact.

Limitations to our study, besides the modest sample size, include the use of CT instead of Magnetic Resonance Imaging to estimate stroke location and the use of the ASPECT score which is a crude measure of stroke volume (18). Also, we did not adjust for stroke volumes but, as stated, excluded significant mass effects, ensuring integrity of brainstem pathways. Lastly, the NPi is an index based on a proprietary algorithm, and although it is commonly used in the clinical setting e.g., (20, 21, 32, 33), it cannot be publicly verified, which limits its intrinsic value to the scientific community (We contacted the manufacturer but were unable to receive information about how the NPi is computed).

On the positive side, all pupillometry data besides the NPi are based on objective and well-known indices (Table 1), and this is one of the few systematic studies investigating cortical modulation of pupillary function in humans. Moreover, we worked within a true-to-life clinical research setting, using noninvasive and easily available tools; we included controls adequately matched in terms of sex, vascular co-morbidity and age (34, 35). Further, we excluded confounding factors such as eye diseases, posterior circulation strokes and intracranial structural pathologies other than ischemic stroke.

Of note, we also introduced a new cortical lesion model in humans (which are very rare for obvious reasons), using strategic infarctions in patients after endovascular thrombectomy for acute ischemic stroke. Given the rapidly increasing use of endovascular stroke therapy (36), this seems to be a feasible way to recruit patients within a comparatively short timeframe and to systematically study the neuronal effects of select cortical lesions in a real-world setting.

## Conclusions

Overall pupillary function is unaffected by prefrontal eye field or insular cortex strokes in humans. Subtle changes, perhaps related to autonomic dysfunction, may still occur immediately after the light reflex when the pupils dilate back to baseline. Replication using a larger sample size is needed to further explore the possible influence of hemispheric lateralization. We suggest that endovascular therapy for acute ischemic stroke due to occlusive large vessel disease may serve as a pragmatic and realistic clinical research model for the study of acquired cortical lesions in humans.

## Author contributions

CP: acquisition of data, analysis and interpretation, writing of the manuscript, critical revision for important intellectual content, approval of final manuscript; PM, JG: acquisition of data, critical revision for important intellectual content, approval of final manuscript; TT, GK: analysis and interpretation, critical revision for important intellectual content, approval of final manuscript; JK: study concept, analysis and interpretation, critical revision for important intellectual content, approval of final manuscript; DK: study concept, acquisition of data, analysis and interpretation, writing of the manuscript, critical revision for important intellectual content, approval of final manuscript.

### Conflict of interest statement

The authors declare that the research was conducted in the absence of any commercial or financial relationships that could be construed as a potential conflict of interest.
